# Tracking Control for Wheeled Mobile Robot Based on Delayed Sensor Measurements

**DOI:** 10.3390/s19235177

**Published:** 2019-11-26

**Authors:** El-Hadi Guechi, Karim Belharet, Sašo Blažič

**Affiliations:** 1 Laboratoire d’Automatique de Skikda (LAS), Faculté de Technologie, Département de Génie Électrique, Université 20 Août 1955, BP 26, Route El-Hadaeik, Skikda 21000, Algeria; e.guechi@univ-skikda.dz; 2 Laboratoire PRISME, HEI Campus Centre, 2 Allée Jean Vaillé, 36000 Châteauroux, France; karim.belharet@yncrea.fr; 3 Faculty of Electrical Engineering, University of Ljubljana, Tržaška 25, 1000 Ljubljana, Slovenia

**Keywords:** delayed sensor measurements, nonholonomic mobile robot, predictor observer, PDC control, trajectory tracking

## Abstract

This paper proposes a novel Takagi-Sugeno fuzzy predictor observer to tackle the problem of the constant and known delay in the measurements. The proposed observer is developed for a trajectory-tracking problem of a wheeled mobile robot where a parallel-distributed compensation control is used to control the robot. The L2-stability of the proposed observer is also proven in the paper. Both, the control and the observer gains are obtained by solving the proposed system of linear matrix inequalities. To illustrate the efficiency of the proposed approach, an experimental comparison with another predictor observer was done.

## 1. Introduction

In several applications of control systems, processing or transmitting information is necessary to make decisions. To determine location or perceive an environment may introduce more or less significant delays [1,2,3,4,5,6]. For example, the measurement of the linear velocity of the vehicle given by a GPS sensor is usually delayed with respect to the real velocity of the vehicle. This is usually caused by a sensor processing delays or momentary sensor outage. Another example is measuring the position of an object or a group of objects with a camera sensor where extensive image processing involving several phases is necessary in order to produce the final result. Such a vision-based sensor introduces a significant delay that usually cannot be ignored in the control law design. It is clear that measurement delays could affect the stability and robustness or at least could lead to a severe degradation of performance of observers and/or control systems [7,8,9]. To tackle the problem of delayed measurements, classically a modified estimator is used that compares each delayed measurement with its corresponding backward time-shifted estimate. The weakness of the modified estimators is that they require a careful adjustment of the gain to ensure their stability, which leads to poor transient responses [10,11,12]. Other alternatives are to use a predictor observer that compares the delayed measurements with the estimated delayed measurements, in order to compensate for the delay. Many authors adopt this approach in their works. In [13], a predictive observer is proposed to solve the problem of landmark measurements obtained with time-delay and it is applied in the case of wheeled mobile robots (WMR). In [14], a nonlinear observer with a chain-like structure is proposed that explicitly reflects and takes into account the magnitude of the output delay. The gains of the observer are computed from the solution of a system of first-order singular partial differential equations. In [15], an observer–predictor methodology to compensate for the delay in the measurements for three classes of systems on Lie groups (left-invariant, right-invariant, and mixed-invariant) is proposed. In [16], a nonlinear observer structure for estimating position, velocity and attitude in a Global Navigation Satellite Systems (GNSS)/Inertial Navigation Systems (INS) with delayed GNSS-measurements is proposed. In [17], an observer for a class of multi-output nonlinear systems with multi-rate sampled and delayed output measurements is designed. This observer can be designed for a specific form of nonlinear systems where the system matrix A is in the Brunovsky form. In [18], an observer is proposed for a wide class of nonlinear systems with both sampled and delayed measurements but its existence is ensured only if several hypotheses are fulfilled. In [19], interval observers for linear systems are proposed where the delay in the measurements is time varying. In [20], the control of a networked system with delayed measurements is treated where the strategy to compensate for the delay is based on using an adaptive Smith predictor. In [21], a generalized observer to tackle the delay problem in the measurements for a specific form of one-sided Lipschitz nonlinear systems is proposed. In [22], a general framework is proposed for the design of attitude estimators by assuming that measurements are delayed. Here, the proposed approach is applied in the case of satellite system where a vector measurement is provided by a magnetometer. In [23], a predictive strategy for maintaining the formation of mobile robots is proposed to solve the problem of communication delays. In [24], a nonlinear predictive observer is proposed to compensate for the delay introduced by a vision-based sensor and the application is trajectory tracking of nonholonomic WMRs.

In the above-cited works, the proposed observers to tackle the problem of the delayed measurements are valid for either linear systems or a specific class of nonlinear systems. The contribution of this paper is that the proposed TS fuzzy predictor observer to compensate for the delay in the outputs can be designed for a large class of nonlinear systems for which an equivalent Takagi-Sugeno (TS) fuzzy model can be determined. Takagi-Sugeno representation can be obtained in a compact region of the state-space for a relatively large class of nonlinear systems including WMRs as will be shown in the paper. It is to be noted that the observer design is done in the framework of the linear matrix inequalities (LMI) meaning that the observer gains are determined solving the system of LMIs [25].

The main contributions of this paper can be summarized as follows:

- the observer is developed for trajectory tracking of a WMR in the case of delayed measurements where the delay is constant and known;
- the stability of the proposed approach is treated formally;
- the proposal of a coordinate transformation for the orientation error which results in an increased feasibility region of the LMI problem and better tracking of the reference trajectory;
- validation of the approach on the real platform of a MIABOT mobile robot.

This paper is organized as follows. Section 2 comprises of four parts. The first one deals with modeling of wheeled mobile robots; in the second one, the PDC control of the WMR is presented; a nonlinear predictor observer from [24] is briefly sketched in the third part; the TS fuzzy predictor observer to compensate for the delay in the measured output is presented in the fourth part. Experimental results are provided in Section 3 to demonstrate the efficiency of the proposed approach.

## 2. Materials and Methods

### 2.1. Kinematic Model of a Wheeled Mobile Robot

In this section, we will briefly present a simple kinematic model of a WMR. This model is used for control purposes in this paper. Very often, a kinematic model is used for control design of a WMR instead of a dynamic one. The main reasons for such a choice are:

- Calculating the control for a kinematic model (speed control) is in general simpler than for a dynamic model (toque control).
- There are no complex geometric or inertial parameters to be identified for a kinematic model.
- Finally, very often (e.g., in the case of miniature mobile robots used in our application), the inertia parameters of the robot are relatively low, while the dynamics of the actuators and the power stage are very fast.

#### 2.1.1. Kinematic Model

**Figure 1:**
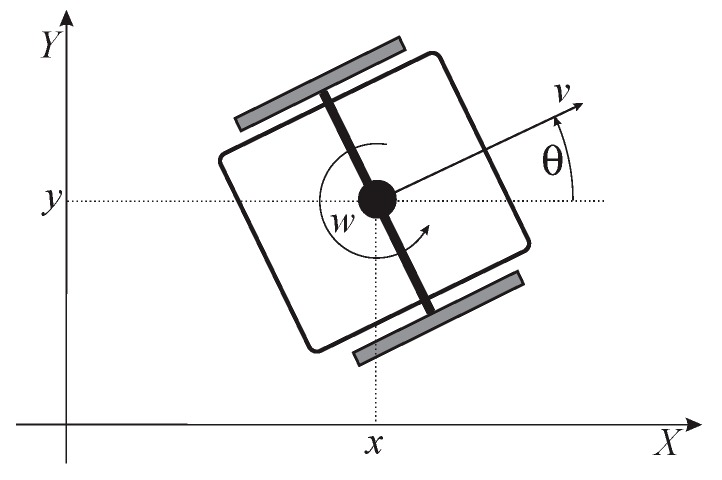
Differentially driven mobile robot with pose and control variables [26].

By taking into account the non-slipping condition, the kinematic model of the WMR in the X-Y plane (see Figure 1) can be written as follows:

(1) $$\begin{array}{l} \dot{x}=v\;\cos\theta \\ \dot{y}=v\;\sin\theta \\ \dot{\theta }=w \end{array}$$

where the considered control inputs of the WMR *v* and *w* are the linear and the angular speed of the robot, respectively. The output variables are *x* and *y* (the robot gravity-center position) and θ (the angle between the speed vector and the *X*-axis, i.e., the robot orientation).

#### 2.1.2. Kinematic Error-Model of Trajectory Tracking

In this section, the well-known error model of the system is derived that is used for the control purposes. The reference trajectory of the robot should be known in advance. It is given as $q_{r}\left(t\right)={\left[\begin{array}{ccc} x_{r}\left(t\right) & y_{r}\left(t\right) & \theta_{r}\left(t\right) \end{array}\right]}^{T}$. By using flatness-based approach [27], the linear reference velocity (denoted by $v_{r}$) and the angular reference velocity (denoted by $w_{r}$) can be obtained from the reference trajectory:

(2) $$\begin{array}{l} v_{r}\left(t\right)=\sqrt{{\dot{x}}_{r}^{2}\left(t\right)+{\dot{y}}_{r}^{2}\left(t\right)}\\ w_{r}\left(t\right)=\frac{{\dot{x}}_{r}\left(t\right){\ddot{y}}_{r}\left(t\right)-{\dot{y}}_{r}\left(t\right){\ddot{x}}_{r}\left(t\right)}{{\dot{x}}_{r}^{2}\left(t\right)+{\dot{y}}_{r}^{2}\left(t\right)} \end{array}$$

**Figure 2:**
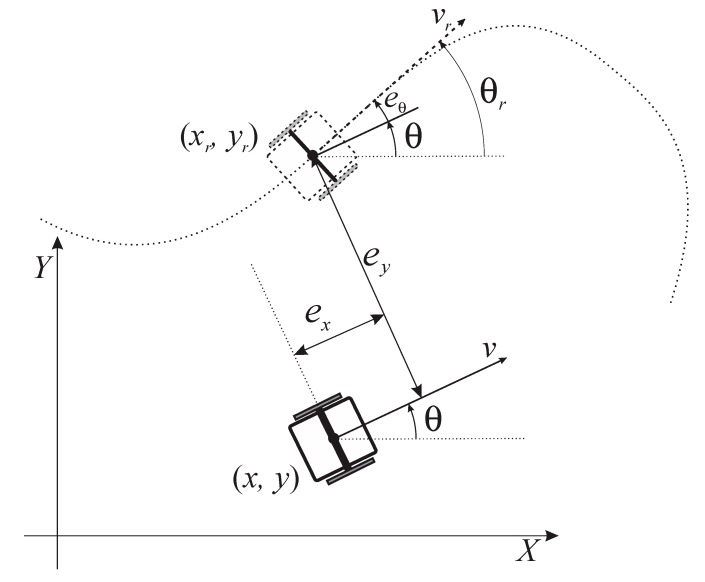
Posture error [28].

Figure 2, illustrates the definition of the posture error $e={\left[\begin{array}{ccc} e_{x} & e_{y} & e_{\theta } \end{array}\right]}^{T}$ expressed in the frame of the real robot. Using the actual posture $q={\left[\begin{array}{ccc} x & y & \theta \end{array}\right]}^{T}$ of the real robot and the reference posture $q_{r}={\left[\begin{array}{ccc} x_{r} & y_{r} & \theta_{r} \end{array}\right]}^{T}$ of a virtual reference robot the following error model can be obtained [28]:

(3) $$\left[\begin{array}{c} e_{x}\\ e_{y}\\ e_{\theta } \end{array}\right]=\left[\begin{array}{ccc} \cos\left(\theta \right) & \sin\left(\theta \right) & 0\\ -\sin\left(\theta \right) & \cos\left(\theta \right) & 0\\ 0 & 0 & 1 \end{array}\right]\left(q_{r}-q\right)$$

From (1) and (3) and assuming that the virtual robot has a kinematic model similar to (1), the posture error model can be written as follows [28]:

(4) $$\left[\begin{array}{c} {\dot{e}}_{x}\\ {\dot{e}}_{y}\\ {\dot{e}}_{\theta } \end{array}\right]=\left[\begin{array}{cc} \cos\left(e_{\theta }\right) & 0\\ \sin\left(e_{\theta }\right) & 0\\ 0 & 1 \end{array}\right]\left[\begin{array}{c} v_{r}\\ w_{r} \end{array}\right]+\left[\begin{array}{cc} -1 & e_{y}\\ 0 & -e_{x}\\ 0 & -1 \end{array}\right]u$$

where $v_{r}$ is the linear reference velocity and $w_{r}$ is the angular reference velocity. The control law is then defined as $u^{T}=\left[\begin{array}{cc} v & w \end{array}\right]$. In [28,29] the authors proposed to set $u=u_{F}+u_{B}$ with $u_{F}={\left[\begin{array}{cc} v_{r}\;\cos\left(e_{\theta }\right) & w_{r} \end{array}\right]}^{T}$ a feed-forward control action vector and $u_{B}={\left[\begin{array}{cc} v_{b} & w_{b} \end{array}\right]}^{T}$ a feedback control action to be defined later. Inserting the control expression into (4), the resulting model is given by:

(5) $$\left[\begin{array}{c} {\dot{e}}_{x}\\ {\dot{e}}_{y}\\ {\dot{e}}_{\theta } \end{array}\right]=\left[\begin{array}{ccc} 0 & w_{r} & 0\\ -w_{r} & 0 & v_{r}\sin c\left(e_{\theta }\right)\\ 0 & 0 & 0 \end{array}\right]\left[\begin{array}{c} e_{x}\\ e_{y}\\ e_{\theta } \end{array}\right]+\left[\begin{array}{cc} -1 & e_{y}\\ 0 & -e_{x}\\ 0 & -1 \end{array}\right]u_{B}$$

where:

(6) $$\sin c\left(\alpha \right)=\left\{ \begin{array}{l} \frac{\sin\left(\alpha \right)}{\alpha }\;\alpha \neq 0\\ 1\;\alpha =0 \end{array}\right.$$

### 2.2. Parallel Distributed Compensation Control of a WMR

In this section, we will first define the control problem. Then we will propose the solution in the form of the Parallel Distributed Compensation (PDC) Control that is based on the Takagi-Sugeno (TS) fuzzy model of a WMR.

#### 2.2.1. Control Problem Statement

The control problem is formulated as follows. We would like to design the control $u_{B}\left(t\right)$ to drive the states of the system (5) to 0 taking into account the bounds for the control and assuming that the initial condition e(0) of the system is in the vicinity of the origin. Euclidean norm of the control vector and the initial state vector, respectively, will be used to denote their size similarly as proposed in [25,30]. There is, however, the problem in the interpretation of the bounds of the control $u_{B}$, and also the size of the set of admissible initial conditions e(0). Just using a norm $\left\|u_{B}\right\|$ is inappropriate because there are different types of variables (linear velocity and angular velocity) in $u_{B}$ and they can be in the different order of magnitude. The same holds for the norm of the initial condition e(0) where there are two spatial components ($e_{x}\left(0\right)$ and $e_{y}\left(0\right)$), and an angular one ($e_{\theta }\left(0\right)$). To solve this problem, we introduce a coordinate transformation on the orientation error $e_{\theta }$ to produce a new error $e_{\theta }^{\prime }$ that is given in the units of distance. Similarly, $w_{b}^{\prime }$ is introduced that is given in the units of linear velocity:

(7) $$\begin{array}{l} e_{\theta }^{\prime }\left(t\right)=k_{t}\;e_{\theta }\left(t\right)\\ w_{b}^{\prime }=k_{t}w_{b} \end{array}$$

where $k_{t}$ is a constant (usually given in m/rad). Its magnitude will be discussed later. Now we have a new tracking error vector $e^{\prime }=\left[\begin{array}{ccc} e_{x} & e_{y} & e_{\theta }^{\prime } \end{array}\right]$ and a new control $u_{B}^{\prime }={\left[\begin{array}{cc} v_{b} & w_{b}^{\prime } \end{array}\right]}^{T}$. Both now include the variables with the same physical meaning, and the norm of these two vectors can now be interpreted easier. We shall also see that there is another important consequence of this new parameter–by properly tuning this parameter the feasibility region of the control problem becomes larger and consequently a faster convergence of the control error is achieved thanks to a better decay rate.

Substituting the Equation (7) into (5), the kinematic error-model of trajectory tracking becomes as follows:

(8) [e˙xe˙ye˙θ′]=[0wr0−wr0vrktsinc(eθ′kt)000][exeyeθ′]+[−1eykt0−exkt0−1][vbwb′]

**The control problem**: We have to design the control $u_{B}^{\prime }$ based on the full state $e^{\prime }$ for the modified system (8) taking into account the limitation on the control action $\left\|u_{B}^{\prime }\right\|<\sigma$ (σ is a constant chosen according to the WMR control capabilities) and assuming $\left\|e^{\prime }\left(0\right)\right\|<\varsigma$ while $k_{t}$ is a free design parameter to be chosen during the control design.

**Implementation of the control**: After obtaining the tracking error e in the original coordinates, the last component of the vector is multiplied by $k_{t}$ to get the tracking error $e^{\prime }$ used in the control law. In the control action $u_{B}^{\prime }$ the last component is divided by $k_{t}$ before applying the control to the WMR.

#### 2.2.2. TS Fuzzy Model of a WMR

The TS models are represented through the following polytopic Form [31]:

(9) $$\begin{array}{l} \dot{\xi }\left(t\right)=\sum_{i=1}^{r}h_{i}\left(z\left(t\right)\right)\left(A_{i}\xi \left(t\right)+B_{i}u\left(t\right)\right)\\ \upsilon \left(t\right)=\sum_{i=1}^{r}h_{i}\left(z\left(t\right)\right)\left(C_{i}\xi \left(t\right)\right) \end{array}$$

where $\xi \left(t\right)\in R^{n}$ is a state vector, $\upsilon \left(t\right)\in R^{p}$ is an output vector and $z\left(t\right)\in R^{q}$ the premise vector depending on the state vector, $A_{i},\;B_{i}\;\mathrm{and}\;C_{i}$ are constant matrices.

The number of rules *r* is related to the number of the nonlinearities of the model as shown later. Finally, the nonlinear weighting functions $h_{i}\left(z\left(t\right)\right)\;\mathrm{with}\;i\in \left\{1,\ldots ,r\right\}$ are all non-negative, with:

$$\sum_{i=1}^{r}h_{i}\left(z\left(t\right)\right)=1$$

For any nonlinear model, one can find an equivalent fuzzy TS model in a compact region of the state space variable using the sector nonlinearity approach, which consists in decomposing each bounded nonlinear term in a convex combination of its bounds.

Analyzing Equations (5) and (8) one can see that the antecedent vector can be chosen as $z^{T}=\left[\begin{array}{ccc} e^{T} & v_{r} & w_{r} \end{array}\right]$ and $z_{T}^{\prime }=\left[\begin{array}{ccc} e^{\prime }{}^{T} & v_{r} & w_{r} \end{array}\right]$, respectively. We will now describe the system (8) in its equivalent TS fuzzy representation (similar to [24]). Note that there are four bounded nonlinear functions in the error model (8): $n_{1}^{\prime }=w_{r}$, $n_{2}^{\prime }=\frac{v_{r}}{k_{t}}\sin c\left(\frac{e_{\theta }^{\prime }}{k_{t}}\right)$, $n_{3}^{\prime }=e_{y}$ and $n_{4}^{\prime }=e_{x}$ leading to $r=2^{4}=16$ fuzzy rules. The TS fuzzy model of a WMR therefore takes the following form:

(10) $$\frac{de^{\prime }\left(t\right)}{dt}={A^{\prime }}_{z^{\prime }}e^{\prime }\left(t\right)+{B^{\prime }}_{z^{\prime }}u_{B}^{\prime }\left(t\right)$$

where $e^{\prime }\left(t\right)$ is the tracking error $\left[\begin{array}{ccc} e_{x} & e_{y} & e_{\theta }^{\prime } \end{array}\right]$ and $u_{B}^{\prime }\left(t\right)$ is the feedback control, with

(11) $${A^{\prime }}_{z^{\prime }}=\sum_{i=1}^{r}{h^{\prime }}_{i}\left(z^{\prime }\left(t\right)\right){A^{\prime }}_{i},\;{B^{\prime }}_{z^{\prime }}=\sum_{i=1}^{r}h_{i}^{\prime }\left(z^{\prime }\left(t\right)\right)B_{i}^{\prime }$$

where the constant matrices of these 16 fuzzy rules are given as:

(12) $$A_{i}^{\prime }=\left[\begin{array}{ccc} 0 & -\varepsilon_{i}^{1}w_{r,\max} & 0\\ \varepsilon_{i}^{1}w_{r,\max} & 0 & \mu_{i}^{\prime }\\ 0 & 0 & 0 \end{array}\right],\;B_{i}^{\prime }=\left[\begin{array}{cc} -1 & \varepsilon_{i}^{3}e_{\max}\\ 0 & \varepsilon_{i}^{4}e_{\max}\\ 0 & -1 \end{array}\right]$$

where $\varepsilon_{i}^{1},\;\varepsilon_{i}^{3},\;\varepsilon_{i}^{4}\;\mathrm{and}\;\mu_{i}^{\prime }$ are constants, computed according to the reference trajectory [32].

#### 2.2.3. PDC Control of a WMR

We will solve this tracking problem by using the PDC control approach on the TS fuzzy model (10). Using the PDC control law:

(13) $$u_{B}^{\prime }\left(t\right)=-\sum_{i=1}^{r}h_{i}^{\prime }\left(z^{\prime }(t)\right)F_{i}^{\prime }e^{\prime }\left(t\right)=-{F^{\prime }}_{z^{\prime }\left(t\right)}e^{\prime }\left(t\right)$$

the following closed-loop system is obtained:

(14) $$\frac{de^{\prime }\left(t\right)}{dt}=\sum_{i=1}^{r}\sum_{j=1}^{r}h_{i}^{\prime }\left(z^{\prime }(t)\right)h_{j}^{\prime }\left(z^{\prime }(t)\right)\left(A_{i}^{\prime }-B_{i}^{\prime }F_{j}^{\prime }\right)e^{\prime }\left(t\right)$$

Note the $z^{\prime }\left(t\right)$ in the subscript of the fuzzy control gain in Equation (13). This means that $z^{\prime }\left(t\right)$ is used in the antecedent of fuzzy rules.

The TS fuzzy model (14) is guaranteed to be stable if the following LMI conditions are satisfied [33]:

(15) $$\left\{ \begin{array}{l} {ϒ}_{ii}<0,\;i=1,2,\ldots ,r\\ \frac{2}{r-1}{ϒ}_{ii}+{ϒ}_{ij}+{ϒ}_{ji}<0,\;i,j=1,2,\ldots ,r,\;i\neq j \end{array}\right.$$

with ${ϒ}_{ij}=XA_{i}^{\prime }{}^{T}+A_{i}^{\prime }X-M_{j}^{\prime }{}^{T}B_{i}^{\prime }{}^{T}-B_{i}^{\prime }M_{j}^{\prime }$ where X>0 is an arbitrary positive definite matrix of dimension 3 × 3, and $M_{i}^{\prime }$ (*i* = 1, …, *r*) are arbitrary matrices of the appropriate dimensions. Then, $F_{i}^{\prime }=M_{i}^{\prime }X^{-1}$ (*i* = 1, …, *r*) are the stabilizing gains.

Our idea is to design the control that guarantees fast convergence of the control errors taking into account the limitation on the control signal and assuming certain bounds on the initial conditions. We therefore introduce a decay rate γ>0 as a control performance index for ensuring a fast convergence to the reference trajectory. This only introduces an extra term γX in the definition of ${ϒ}_{ij}$ (to make description more clear a new variable ${ϒ}_{ij}^{\gamma }$ is introduced).

The system of control-related LMIs is completed by adding:

(16) $$X\geq \varsigma^{2}I,\;\left[\begin{array}{cc} X & M_{i}^{\prime }{}^{T}\\ M_{i}^{\prime } & \sigma^{2}I \end{array}\right]\geq 0$$

which guarantees the boundedness of the control signals $\left\|u_{B}^{\prime }\right\|<\sigma$ in the case of sufficiently small initial conditions on the state $\left\|e^{\prime }\left(0\right)\right\|<\varsigma$ [25].

The control design is therefore formulated as a generalized eigenvalue problem where the decay rate is optimized to make the closed-loop system as fast as possible respecting the given limitations:

(17) $$\begin{array}{l} \underset{X,\;M_{i}\left(i=1,\cdots ,r\right)}{\mathrm{Maximize}}\;\gamma \;\mathrm{subject}\;\mathrm{to}\\ \left\{ \begin{array}{l} {ϒ}_{ii}^{\gamma }<0,\;i=1,2,\ldots ,r\\ \frac{2}{r-1}{ϒ}_{ii}^{\gamma }+{ϒ}_{ij}^{\gamma }+{ϒ}_{ji}^{\gamma }<0,\;i,j=1,2,\ldots ,r,\;i\neq j\\ X\geq \varsigma^{2}I,\;\left[\begin{array}{cc} X & M_{i}^{\prime }{}^{T}\\ M_{i}^{\prime } & \sigma^{2}I \end{array}\right]\geq 0 \end{array}\right. \end{array}$$

with ${ϒ}_{ij}^{\gamma }=XA_{i}^{\prime }{}^{T}+A_{i}^{\prime }X-M_{j}^{\prime }{}^{T}B_{i}^{\prime }{}^{T}-B_{i}^{\prime }M_{j}^{\prime }+\gamma X$.

If we get the solution of the above generalized eigenvalue problem (with positive value of γ), the closed-loop system with control gains given by $F_{i}^{\prime }=M_{i}^{\prime }X^{-1}$ (*i* = 1, …, *r*) is stable and γ serves as a decay rate of the control errors.

Until now we have not discussed how the constant $k_{t}$ > 0 is chosen. It should be chosen to make the performance of the original system (5) as good as possible in terms of both the error convergence and the control actions. However, as a rule of thumb the following formula can be used for the selection of $k_{t}$:

(18) $$k_{t}=\frac{d_{\max}}{\left|e_{\theta_{\max}}\right|}$$

where $d_{\max}$ is the maximum allowable value of the initial distance from the reference trajectory and $e_{\theta_{\max}}$ is the maximum allowable initial orientation error. By selecting $k_{t}$ according to (18), the contribution of the orientation error ($e_{\theta }$) is equilibrated to the “distance” error ($\sqrt{e_{x}^{2}+e_{y}^{2}}$) in $e^{\prime }$.

### 2.3. Nonlinear Predictor Observer

In this section we introduce the solution for the trajectory tracking problem of a WMR, based on PDC control and nonlinear predictor observer, in the case of the delayed measurements proposed in [24]. In Section 2.3 and Section 2.4, the original TS fuzzy model of the tracking problem (5) will be treated

(19) $$\frac{de\left(t\right)}{dt}=A_{z}e\left(t\right)+B_{z}u_{B}\left(t\right)$$

The rationale behind this is to simplify the notation (primes are omitted), and more importantly to show that different scaling factors can be used in the controller and the observer case (which is a natural consequence of the different error sources). So, the nonlinear predictor observer proposed in [24], is given by the following formula:

(20) $$\begin{array}{l} \dot{\hat{x}}\left(t\right)=v\left(t\right)\cos\left(\hat{\theta }\left(t\right)\right)-L_{1}\left(x\left(t-\tau \right)-\hat{x}\left(t-\tau \right)\right)\\ \dot{\hat{y}}\left(t\right)=v\left(t\right)\sin\left(\hat{\theta }\left(t\right)\right)-L_{2}\left(y\left(t-\tau \right)-\hat{y}\left(t-\tau \right)\right)\\ \dot{\hat{\theta }}\left(t\right)=w\left(t\right)-L_{3}\left(\theta \left(t-\tau \right)-\hat{\theta }\left(t-\tau \right)\right) \end{array}.$$

where *L*_1_, *L*_2_, *L*_3_ are constant gains, and *τ* is the value of the measurement delay assumed to be constant. The observer (20) is asymptotically stable if the gains *L_i_* are such that $L_{i}\tau <\pi /2\;\mathrm{for}\;i\in \left\{1,2,3\right\}$. The control law will now use estimated error instead of the actual one:

(21) $$u_{B}(t)=-F_{\hat{z}(t)}\hat{e}(t)$$

**Figure 3:**
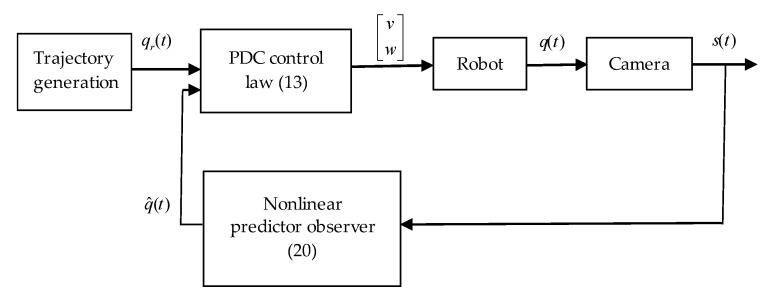
Block diagram of the whole-closed loop system with nonlinear observer.

The whole closed loop control is shown in Figure 3, where we also introduced the measured pose of the robot s(t). Since the pose is obtained by a digital camera, the measurement is always obtained with a certain delay:

(22) $$s(t)=\left[\begin{array}{c} s_{x}(t)\\ s_{y}(t)\\ s_{\theta }(t) \end{array}\right]=q(t-\tau )$$

where the delay τ will be treated as constant. The measurement is also corrupted due to optical errors, imperfect-observing conditions etc. All these errors contribute to the measurement error which will be discussed later.

### 2.4. TS Fuzzy Predictor Observer

In this section, we propose a new approach based on the PDC control law and TS fuzzy predictor observer, to solve the problem of the trajectory tracking, in the case of delayed measurements, where the delay is assumed constant. The TS fuzzy model of the WMR with delayed measurements is given by extending the original TS fuzzy model (19) with an output υ(t) composed of delayed states:

(23) $$\begin{array}{l} \frac{de\left(t\right)}{dt}=A_{z}e\left(t\right)+B_{z}u_{B}\left(t\right)\\ \upsilon \left(t\right)=e\left(t-\tau \right) \end{array}$$

where υ(t)=e(t−τ) is obtained using Equation (3) and taking into account Equation (22):

(24) $$\upsilon \left(t\right)=e\left(t-\tau \right)=\left[\begin{array}{ccc} \cos\left(s_{\theta }\left(t\right)\right) & \sin\left(s_{\theta }\left(t\right)\right) & 0\\ -\sin\left(s_{\theta }\left(t\right)\right) & \cos\left(s_{\theta }\left(t\right)\right) & 0\\ 0 & 0 & 1 \end{array}\right]\left(q_{r}\left(t-\tau \right)-s\left(t\right)\right)$$

The same control law (21) as in the previous section is used. Consider the following TS fuzzy predictor observer:

(25) $$\frac{d\hat{e}\left(t\right)}{dt}=A_{\hat{z}}\hat{e}\left(t\right)+B_{\hat{z}}u_{B}\left(t\right)-\kappa_{\hat{z}}\left(\upsilon \left(t\right)-\hat{e}\left(t-\tau \right)\right)$$

with $\hat{e}\left(t\right)$ the estimated state, and τ a constant delay in the output.

The TS fuzzy tracking error model (23) can be written in the following manner:

(26) $$\dot{e}\left(t\right)=A_{\hat{z}}e\left(t\right)+B_{\hat{z}}u_{B}\left(t\right)+I\varpi \left(t\right)$$

where:

- *I* is the identity matrix;
- $\varpi \left(t\right)=\left(A_{z}-A_{\hat{z}}\right)e\left(t\right)+\left(B_{z}-B_{\hat{z}}\right)u_{B}\left(t\right)$ acts as a disturbance.

In order to avoid loss of controllability, the TS fuzzy model of the WMR is obtained by assuming the error vector e(t) is bounded [24]. The control $u_{B}\left(t\right)$ is determined from control gains that are obtained solving the generalized eigenvalue problem (17). In the LMI (17) there is a condition that the robot control does not exceed the maximum physical value allowed. The error vector e(t) is bounded by assumption, the control $u_{B}\left(t\right)$ is bounded if a feasible solution of the problem in (17) is found, the boundedness of $\left(A_{z}-A_{\hat{z}}\right)$ and $\left(B_{z}-B_{\hat{z}}\right)$ comes from the stability of the observer; and therefore the disturbance ϖ(t) is bounded. The experimental results presented in Section 3 substantiate the theoretical part.

We define the estimated error $\tilde{e}\left(t\right)=e\left(t\right)-\hat{e}\left(t\right)$. Subtracting (25) from (26), the dynamics of the estimated error is obtained:

(27) $$\frac{d\tilde{e}\left(t\right)}{dt}=A_{\hat{z}}\tilde{e}\left(t\right)+\kappa_{\hat{z}}\tilde{e}\left(t-\tau \right)+I\varpi \left(t\right).$$

Next, the main result of this paper is derived. The *L*_2_-stability of the estimated error dynamics (27) is proven. The effect of the disturbance term ϖ is attenuated in a similar manner as proposed in [34] for the case of non-delayed measurements.

**Theorem** **1.** *The dynamics of the estimated error (27) are stable if there exist common matrices $P_{1},\;P_{2},\;R>0$, $M_{i}\left\{i=1,\ldots ,r\right\}$ of dimension 3 × 3, and a positive constant ρ such that:*

(28) $$\left[\begin{array}{cccc} X_{11} & X_{12} & 0 & 0\\ \left(*\right) & X_{22} & \tau M_{i} & P_{2}\\ \left(*\right) & \left(*\right) & -\tau R & 0\\ \left(*\right) & \left(*\right) & \left(*\right) & -\rho^{2}I_{n} \end{array}\right]<0$$

*where*

- $X_{11}=A_{i}^{T}P_{2}+P_{2}A_{i}+M_{i}^{T}+M_{i}+I_{n}$;
- $X_{12}=A_{i}^{T}P_{2}+M_{i}^{T}+P_{1}-P_{2}$;
- $X_{22}=-P_{2}-P_{2}^{T}+I_{n}+\tau R$.

The observer gains $\kappa_{i}$ are calculated according to:

(29) $$\kappa_{i}=P_{2}^{-1}M_{i}\left(i=1,\cdots ,r\right)$$

Moreover, the *L*_2_-norm of the extended estimated error is upper bounded:

(30) $${\left\|\begin{array}{c} \tilde{e}\\ \dot{\tilde{e}} \end{array}\right\|}_{2}<\rho {\left\|\varpi \right\|}_{2}$$

In order to minimize the norm of the error, we should find the minimum value of ρ that still satisfies (28).

**Proof of Theorem** **1.** Using the Leibniz formula given by the following equation.

(31) $$\tilde{e}\left(t-\tau \right)=\tilde{e}\left(t\right)-\int_{t-\tau }^{t}\dot{\tilde{e}}\left(s\right)ds$$

The estimated error given by the Equation (27) becomes:

(32) $$\frac{d\tilde{e}\left(t\right)}{dt}=\left(A_{\hat{z}}+\kappa_{\hat{z}}\right)\tilde{e}\left(t\right)-\kappa_{\hat{z}}\int_{t-\tau }^{t}\dot{\tilde{e}}\left(s\right)ds+I\varpi \left(t\right)$$

The system presented by the Equation (32) can be rewritten in a descriptor form as follows:

(33) $$\left\{ \begin{array}{l} \dot{\tilde{e}}(t)=\chi \left(t\right)\\ 0=-\chi (t)+\left(A_{\hat{z}}+\kappa_{\hat{z}}\right)\tilde{e}\left(t\right)-\kappa_{\hat{z}}\int_{t-\tau }^{t}\dot{\tilde{e}}\left(s\right)ds+I_{n}\varpi \left(t\right) \end{array}\right.$$

Introducing a constant matrix *E* and an extended estimated error $\bar{e}$

$$\begin{array}{l} E=\left[\begin{array}{cc} I_{n} & 0\\ 0 & 0 \end{array}\right]\\ \bar{e}\left(t\right)={\left[\begin{array}{cc} \tilde{e}\left(t\right) & \dot{\tilde{e}}\left(t\right) \end{array}\right]}^{T} \end{array}$$

We can rewrite Equation (33) in a matrix form

(34) $$E\dot{\bar{e}}\left(t\right)=\left[\begin{array}{cc} 0 & I_{n}\\ A_{\hat{z}}+\kappa_{\hat{z}} & -I_{n} \end{array}\right]\bar{e}\left(t\right)-\left[\begin{array}{c} 0\\ \kappa_{\hat{z}} \end{array}\right]\int_{t-\tau }^{t}\dot{\tilde{e}}\left(s\right)ds+\left[\begin{array}{c} 0\\ I_{n} \end{array}\right]\varpi \left(t\right)$$

Now, we consider the following Lyapunov–Krasovskii functional [35]

(35) $$V\left(t\right)={\bar{e}}^{T}\left(t\right)EP\bar{e}\left(t\right)+\int_{-\tau }^{0}\int_{t+\theta }^{t}{\dot{\tilde{e}}}^{T}\left(s\right)R\dot{\tilde{e}}\left(s\right)dsd\theta$$

with

$$\begin{array}{l} P=\left[\begin{array}{cc} P_{1} & 0\\ P_{2} & P_{3} \end{array}\right]\\ EP=P^{T}E \end{array}$$

where *P*_1_, *P*_2_, *P*_3_ are 3×3 symmetric positive definite matrices.

The derivative of the double integral in V(t) is obtained by using the Leibniz integral rule similarly as in [36]:

(36) $$\frac{dV\left(t\right)}{dt}={\dot{\bar{e}}}^{T}\left(t\right)EP\bar{e}\left(t\right)+{\bar{e}}^{T}\left(t\right)EP\dot{\bar{e}}\left(t\right)+\tau {\dot{\tilde{e}}}^{T}\left(t\right)R\dot{\tilde{e}}\left(t\right)-\int_{t-\tau }^{t}{\dot{\tilde{e}}}^{T}\left(s\right)R\dot{\tilde{e}}\left(s\right)ds$$

Substituting the descriptor model given by (34) into (36), the derivative of V(t) becomes

(37) $$\frac{dV\left(t\right)}{dt}={\bar{e}}^{T}\left(t\right)\psi \left(t\right)\bar{e}\left(t\right)+\varphi \left(t\right)+\tau {\dot{\tilde{e}}}^{T}\left(t\right)R\dot{\tilde{e}}\left(t\right)-\int_{t-\tau }^{t}{\dot{\tilde{e}}}^{T}\left(s\right)R\dot{\tilde{e}}\left(s\right)ds$$

where the following matrices have been introduced:

$$\psi \left(t\right)={\left[\begin{array}{cc} 0 & I_{n}\\ A_{\hat{z}}+\kappa_{\hat{z}} & -I_{n} \end{array}\right]}^{T}P+P^{T}\left[\begin{array}{cc} 0 & I_{n}\\ A_{\hat{z}}+\kappa_{\hat{z}} & -I_{n} \end{array}\right]$$

$$\varphi \left(t\right)=\varphi_{1}\left(t\right)+\varphi_{2}\left(t\right)\;\mathrm{with}\;\left\{ \begin{array}{l} \varphi_{1}\left(t\right)=2{\bar{e}}^{T}P^{T}\left[\begin{array}{c} 0\\ I_{n}\varpi \left(t\right) \end{array}\right]\\ \varphi_{2}\left(t\right)=-2{\bar{e}}^{T}P^{T}\left[\begin{array}{c} 0\\ \kappa_{\hat{z}} \end{array}\right]\int_{t-\tau }^{t}\dot{\tilde{e}}\left(s\right)ds \end{array}\right.$$

In order to find the upper bound of $\varphi_{2}\left(t\right)$, we use the following lemma: For all positive definite matrices R(n×n) and for all vectors $a,b\in {\mathbb{R}}^{n}$, the following inequality is always satisfied:

$$\pm 2a^{T}b\leq a^{T}R^{-1}a+b^{T}Rb.$$

Applying this lemma to the term $\varphi_{2}\left(t\right)$ with $a^{T}={\bar{e}}^{T}P^{T}\left[\begin{array}{c} 0\\ \kappa_{\hat{z}} \end{array}\right]$ and $b=\dot{\tilde{e}}\left(s\right)$, the following inequality is obtained:

(38) $$\begin{array}{c} \varphi_{2}\left(t\right)\leq \int_{t-\tau }^{t}\left\{{\bar{e}}^{T}P^{T}\left[\begin{array}{c} 0\\ \kappa_{\hat{z}} \end{array}\right]\;R^{-1}\;{\left[\begin{array}{c} 0\\ \kappa_{\hat{z}} \end{array}\right]}^{T}P\bar{e}+{\dot{\tilde{e}}}^{T}\left(s\right)R\;\dot{\tilde{e}}\left(s\right)\right\}ds\\ \leq \tau {\bar{e}}^{T}P^{T}\left[\begin{array}{c} 0\\ \kappa_{\hat{z}} \end{array}\right]\;R^{-1}\;{\left[\begin{array}{c} 0\\ \kappa_{\hat{z}} \end{array}\right]}^{T}P\bar{e}+\int_{t-\tau }^{t}{\dot{\tilde{e}}}^{T}\left(s\right)R\;\dot{\tilde{e}}\left(s\right) \end{array}$$

In [34] it was proven that (30) is achieved if V(t) satisfies the condition:

(39) $$\frac{dV\left(t\right)}{dt}+{\bar{e}}^{T}\left(t\right)\bar{e}\left(t\right)-\rho^{2}\varpi^{T}\left(t\right)\varpi \left(t\right)<0$$

Substituting (38) and (37) into (39), the stability condition of the TS fuzzy predictor observer becomes:

(40) $${\bar{e}}^{T}\left(t\right)\psi_{0}\left(t\right)\bar{e}\left(t\right)+2{\bar{e}}^{T}P^{T}\left[\begin{array}{c} 0\\ I_{n}\varpi \left(t\right) \end{array}\right]-\rho^{2}\varpi^{T}\left(t\right)\varpi \left(t\right)<0$$

with

$$\psi_{0}\left(t\right)=\psi \left(t\right)+I_{2n}+\left[\begin{array}{cc} 0 & 0\\ 0 & \tau R \end{array}\right]+\tau {\bar{e}}^{T}P^{T}\left[\begin{array}{c} 0\\ \kappa_{\hat{z}} \end{array}\right]\;R^{-1}\;{\left[\begin{array}{c} 0\\ \kappa_{\hat{z}} \end{array}\right]}^{T}P\bar{e}.$$

Representing (40) in the matrix form, the following inequality can be written:

(41) $${\left[\begin{array}{c} \bar{e}\left(t\right)\\ \varpi \left(t\right) \end{array}\right]}^{T}\left[\begin{array}{cc} \psi_{0}(t) & P\left[\begin{array}{c} 0\\ I_{n} \end{array}\right]\\ \left(*\right) & -\rho^{2} \end{array}\right]\left[\begin{array}{c} \bar{e}\left(t\right)\\ \varpi \end{array}\right]<0$$

Applying Schur’s formula to the term $\psi_{0}(t)$ the inequality (41) can be rewritten as follows:

(42) $$\left[\begin{array}{ccc} \psi \left(t\right)+\left[\begin{array}{cc} 0 & 0\\ 0 & \tau R \end{array}\right]+I_{6} & \tau P\left[\begin{array}{c} 0\\ \kappa_{i} \end{array}\right] & P\left[\begin{array}{c} 0\\ I_{3} \end{array}\right]\\ \left(*\right) & -\tau R & 0\\ \left(*\right) & 0 & -\rho^{2}I_{3} \end{array}\right]<0\;i=1\ldots r$$

where the following matrices are used:

- $I_{n}$ is an n×n identity matrix;
- *R* is 3×3 symmetric positive definite matrix.

The inequality (42) is not in the LMI form because it contains a product of *P* and $\kappa_{i}$, which are both variables that we search for. One way to obtain an LMI formulation of our problem is to choose *P*_3_ equal to *P*_2_ and to introduce $M_{i}=P_{2}\kappa_{i}$ (*i* = 1, …, *r*). In this case, the inequality (42) can be written in the LMI form represented in (28). □

**Figure 4:**
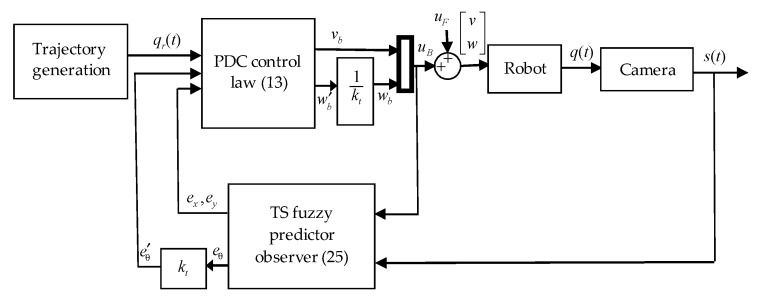
Block diagram of the whole closed loop system with TS fuzzy predictor observer.

In our case of the trajectory tracking with a constant delay in measurements, the fulfilment of the LMI conditions given by the theorem results in the asymptotic stability of the proposed TS fuzzy predictor observer (in the absence of measurement noise). When some noise is added, its effect is attenuated in a similar way to the effect of ϖ. Consequently, the *L*_2_-stability of the observer is retained and the errors in the system are bounded in their *L*_2_-norm. To show the efficiency of the proposed observer some experimental results are given which is the aim of the next section. The block diagram of the whole closed loop control system using PDC control and TS fuzzy predictor observer (25) is shown in Figure 4.

## 3. Results

In order to illustrate the efficiency of the proposed TS fuzzy predictor observer, an experimental study and the comparison with the observer presented in [24], were performed. The proposed approach was applied to the trajectory tracking for a MIABOT mobile robot. The robot pose is measured via Basler (A311fc) camera whose frame rate is 30 images per second. The measurement delay is due to image processing on the camera itself, communication, and processing on the desktop computer. The whole delay from the time the image was taken to the time the robot pose is available was measured experimentally to be 66 ms (with slight variations). The delay was assumed constant meaning that the variations were not taken into account. Rather, they served to show the robustness of the algorithm to slight variations of the delay.

**Figure 5:**
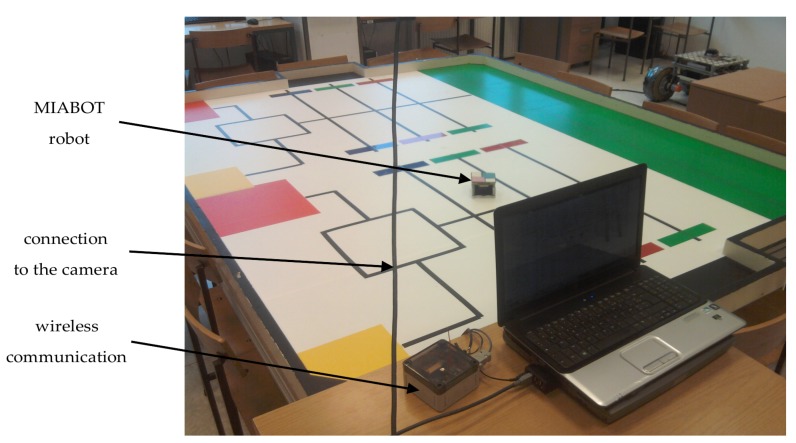
MIABOT mobile robot platform.

The control is sent to the robot via wireless communication. The control code is programmed in C++ which is adequate for the real-time application. The MIABOT mobile robot platform is shown in Figure 5.

In the following experimental study, we will use the visual-based soft sensor that provides the measured orientation in degrees. The latter could have been converted to radians but we chose not to do this in order to show a potential problem that is encountered when the error states are different for several orders of magnitude (the same problem would arise if orientation is given in radians but the position measurements are either very high or very low in amplitude).

It is very easy to obtain the control gains by solving the LMIs for the system that operates with degrees (some scalar gains are of course different from the case where the measurements are in radians). It turns out that the approach is not robust if error normalization given by (7) is not applied. This is why we will also show the experimental results with and without this error normalization (or coordinate transformation) implemented.

The reference trajectory has a shape of the number eight. The robot reference initial and final positions are the same: *x_i_* = *x_f_* = 0.4 m; *y_i_* = *y_f_* = 0.7 m; *θ_i_* = −90°.

### 3.1. The Use of Original Tracking Error e in the Control Law

In this section, the control law will be obtained based on the original error model (5). Solving the generalized eigenvalue minimization problem (using MATLAB and Robust Control Toolbox) given in (17) with $\sigma^{2}=5$ and $\varsigma^{2}=0.01$, we obtain the decay rate of γ=0.01 and the following gains of the TS fuzzy model:

$$F_{1}=\left[\begin{array}{ccc} -13.6117 & -3.0274 & -0.1045\\ 2.4223 & -16.1618 & -0.4729 \end{array}\right],\;F_{2}=\left[\begin{array}{ccc} -13.6018 & -3.0778 & -0.1062\\ 2.2274 & -15.0246 & -0.4356 \end{array}\right]$$

$$F_{3}=\left[\begin{array}{ccc} -13.6041 & -3.0765 & -0.1059\\ 2.4554 & -16.1550 & -0.4727 \end{array}\right],\;F_{4}=\left[\begin{array}{ccc} -13.5944 & -3.1249 & -0.1075\\ 2.2558 & -15.0220 & -0.4355 \end{array}\right]$$

$$F_{5}=\left[\begin{array}{ccc} -13.6414 & -3.0305 & -0.1050\\ 2.7587 & -14.8676 & -0.5096 \end{array}\right],F_{6}=\left[\begin{array}{ccc} -13.6378 & -2.8879 & -0.1010\\ 2.6482 & -14.5982 & -0.5056 \end{array}\right]$$

$$F_{7}=\left[\begin{array}{ccc} -13.6327 & -3.0882 & -0.1068\\ 2.8058 & -14.8562 & -0.5092 \end{array}\right],\;F_{8}=\left[\begin{array}{ccc} -13.6294 & -2.9385 & -0.1026\\ 2.6896 & -14.5876 & -0.5053 \end{array}\right]$$

$$F_{9}=\left[\begin{array}{ccc} -13.6039 & 3.0772 & 0.1059\\ -2.4561 & -16.1557 & -0.4727 \end{array}\right],\;F_{10}=\left[\begin{array}{ccc} -13.5942 & 3.1257 & 0.1076\\ -2.2566 & -15.0233 & -0.4356 \end{array}\right]$$

$$F_{11}=\left[\begin{array}{ccc} -13.6116 & 3.0282 & 0.1045\\ -2.4230 & -16.1626 & -0.4729 \end{array}\right],\;F_{12}=\left[\begin{array}{ccc} -13.6017 & 3.0786 & 0.1062\\ -2.2283 & -15.0258 & -0.4357 \end{array}\right]$$

$$F_{13}=\left[\begin{array}{ccc} -13.6325 & 3.0891 & 0.1068\\ -2.8067 & -14.8560 & -0.5092 \end{array}\right],\;F_{14}=\left[\begin{array}{ccc} -13.6292 & 2.9394 & 0.1027\\ -2.6903 & -14.5874 & -0.5053 \end{array}\right]$$

$$F_{15}=\left[\begin{array}{ccc} -13.6413 & 3.0315 & 0.1050\\ -2.7596 & -14.8674 & -0.5096 \end{array}\right],\;F_{16}=\left[\begin{array}{ccc} -13.6377 & 2.8887 & 0.1011\\ -2.6490 & -14.5980 & -0.5056 \end{array}\right]$$

**Figure 6:**
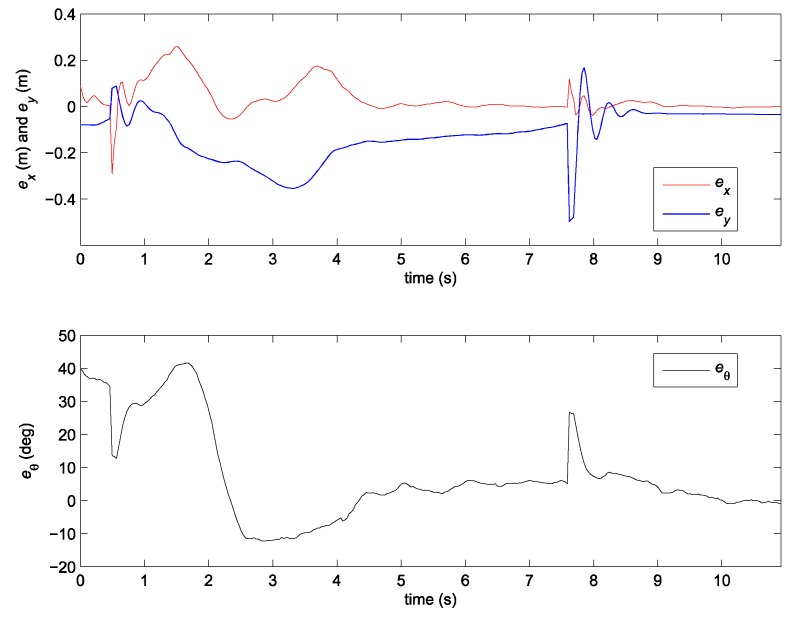
Error of the robot posture in the case of the nonlinear predictor observer (20).

**Figure 7:**
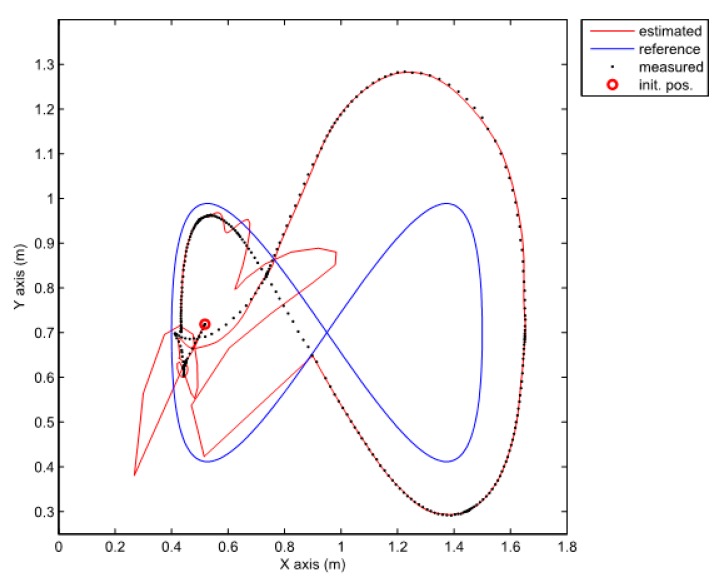
Comparison of the robot trajectories in the case of the nonlinear predictor observer (20).

The gains of the nonlinear predictor observer (20) are chosen inside the stability region and manually tuned to obtain the best tracking possible ($L_{1}=12,\;L_{2}=12,\;L_{3}=5$). The results of the experiment are given in Figure 6 and Figure 7.

Solving the generalized eigenvalue problem given in (28) with τ = 66 ms and *ρ*^2^ = 5, the gains of the TS fuzzy predictor observer (25), $\kappa_{i}=\left(i=1,\ldots ,16\right)$ (see Section 2.4) are as follows:

$$\kappa_{1}=\kappa_{2}=\kappa_{3}=\kappa_{4}=\left[\begin{array}{ccc} -2.2586 & -2.5075 & 0.0027\\ 2.5120 & -2.2606 & 0.0001\\ 0.0028 & -0.0084 & -2.2332 \end{array}\right]$$

$$\kappa_{5}=\kappa_{6}=\kappa_{7}=\kappa_{8}=\left[\begin{array}{ccc} -2.2586 & -2.5075 & 0.0027\\ 2.5120 & -2.2614 & -0.0022\\ 0.0028 & -0.7357 & -2.2324 \end{array}\right]$$

$$\kappa_{9}=\kappa_{10}=\kappa_{11}=\kappa_{12}=\left[\begin{array}{ccc} -2.2586 & 2.5082 & -0.0027\\ -2.5126 & -2.2606 & 0.0001\\ -0.0028 & -0.0084 & -2.2332 \end{array}\right]$$

$$\kappa_{13}=\kappa_{14}=\kappa_{15}=\kappa_{16}=\left[\begin{array}{ccc} -2.2586 & 2.5082 & -0.0027\\ -2.5126 & -2.2614 & -0.0022\\ -0.0028 & -0.7357 & -2.2324 \end{array}\right]$$

**Figure 8:**
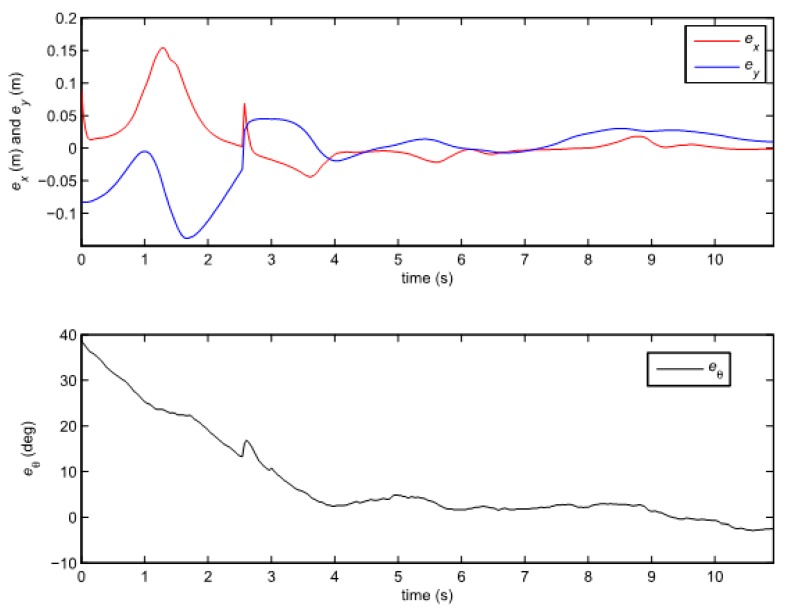
Error of the robot posture in the case of the TS fuzzy predictor observer (25).

**Figure 9:**
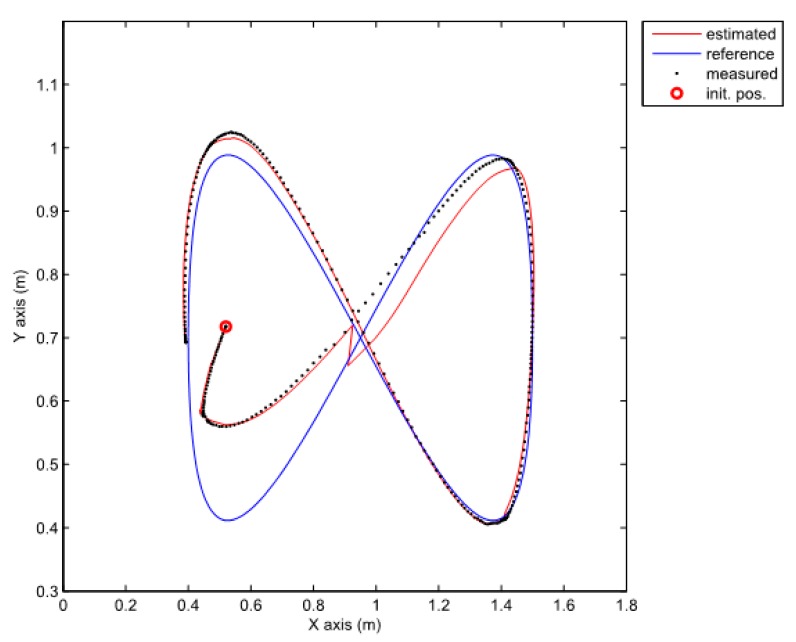
Comparison of the robot trajectories in the case of the TS fuzzy predictor observer (25).

Note that the gains of the observer are the same in all the experiments with TS fuzzy predictor observer. The results of the experiment are given in Figure 8 and Figure 9.

Although the stability of the observer (20) is proven in [24], the stability of the whole closed loop (together with the PDC controller) is not guaranteed. Consequently, the performance of the system may decrease severely to the point where stability is lost. The results in Figure 6 and Figure 7 therefore show bad tracking of the reference trajectory. In the studied case, this phenomenon happens because of the bad scaling of the error states. This problem will be avoided by using the normalization of the error states as illustrated later.

Since the normalization of the states is not performed in the PDC control law, the approach with TS fuzzy predictor observer also suffers from the problems of the bad scaling, but the results are considerably better than in the previous case as shown in Figure 8 and Figure 9. The results will improve by introducing simple transformation (7) as shown in the next section.

### 3.2. The Use of a Modified Tracking Error $e^{\prime }$ in the Control Law

In this section the modified or normalized error $e^{\prime }$ defined in (7) is used and the PDC controller is designed based on the modified error model given by (8). Solving the generalized eigenvalue minimization problem given in (17), with $k_{t}=\frac{\varsigma \sqrt{2}}{90}=0.0016$ given by Equation (18), $\sigma^{2}=5$ and $\varsigma^{2}=0.01$, we obtain the decay rate of γ=0.071 and the following gains of the TS fuzzy model:

$$F_{1}^{\prime }=\left[\begin{array}{ccc} -11.9209 & 0.6100 & -2.1584\\ 0.9756 & -0.1704 & -0.3396 \end{array}\right],\;F_{2}^{\prime }=\left[\begin{array}{ccc} -11.3976 & 0.5437 & -2.6372\\ 0.1019 & -0.1765 & -0.3194 \end{array}\right]$$

$$F_{3}^{\prime }=\left[\begin{array}{ccc} -11.5554 & -0.0934 & -3.9787\\ -1.1761 & -0.1701 & -0.3374 \end{array}\right],\;F_{4}^{\prime }=\left[\begin{array}{ccc} -11.3250 & 0.1357 & -3.3728\\ -0.1010 & -0.1763 & -0.3189 \end{array}\right]$$

$$F_{5}^{\prime }=\left[\begin{array}{ccc} -12.2533 & 2.2080 & 2.3475\\ -1.3187 & -9.7974 & -18.8891 \end{array}\right],F_{6}^{\prime }=\left[\begin{array}{ccc} -12.1098 & 0.9570 & 0.1964\\ 0.0484 & -9.4669 & -18.6382 \end{array}\right]$$

$$F_{7}^{\prime }=\left[\begin{array}{ccc} -10.7419 & -3.5580 & -9.3925\\ 6.0004 & -9.8887 & -16.9853 \end{array}\right],\;F_{8}^{\prime }=\left[\begin{array}{ccc} -11.8914 & 0.7235 & -1.2229\\ 0.8868 & -9.4734 & -18.5767 \end{array}\right]$$

$$F_{9}^{\prime }=\left[\begin{array}{ccc} -11.5543 & 0.0935 & 3.9805\\ 1.1738 & -0.1700 & -0.3374 \end{array}\right],\;F_{10}^{\prime }=\left[\begin{array}{ccc} -11.3241 & -0.1321 & 3.3796\\ 0.0997 & -0.1763 & -0.3189 \end{array}\right]$$

$$F_{11}^{\prime }=\left[\begin{array}{ccc} -11.9207 & -0.6100 & 2.1599\\ -0.9776 & -0.1704 & -0.3397 \end{array}\right],\;F_{12}^{\prime }=\left[\begin{array}{ccc} -11.3970 & -0.5401 & 2.6441\\ -0.1032 & -0.1765 & -0.3194 \end{array}\right]$$

$$F_{13}^{\prime }=\left[\begin{array}{ccc} -10.7396 & 3.5584 & 9.3985\\ -6.0054 & -9.8907 & -16.9827 \end{array}\right],\;F_{14}^{\prime }=\left[\begin{array}{ccc} -11.8913 & -0.7225 & 1.2249\\ -0.8880 & -9.4734 & -18.5766 \end{array}\right]$$

$$F_{15}^{\prime }=\left[\begin{array}{ccc} -12.2534 & -2.2078 & -2.3465\\ 1.3181 & -9.7975 & -18.8893 \end{array}\right],\;F_{16}^{\prime }=\left[\begin{array}{ccc} -12.1098 & -0.9559 & -0.1941\\ -0.0496 & -9.4670 & -18.6382 \end{array}\right]$$

**Figure 10:**
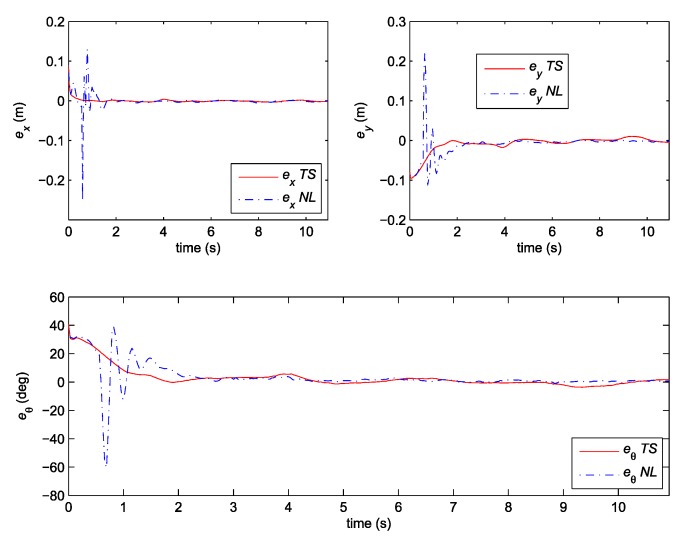
Posture error of the robot: Comparison between TS and NL observer.

**Figure 11:**
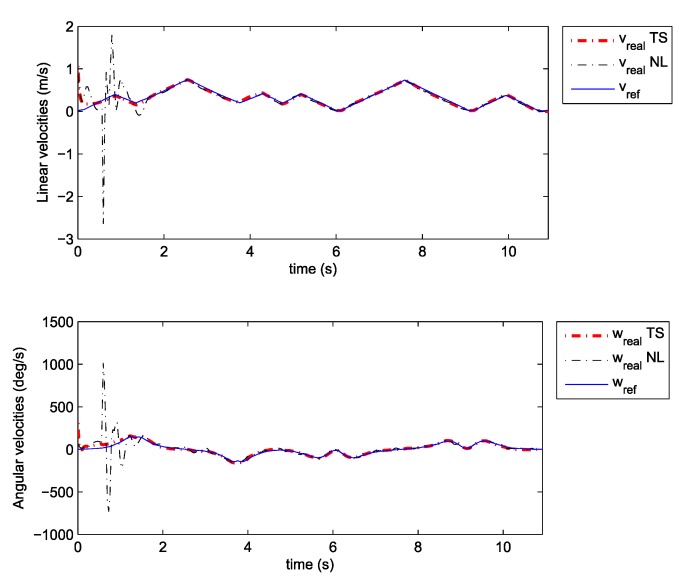
Velocities tracking: Comparison between TS and NL observer.

**Figure 12:**
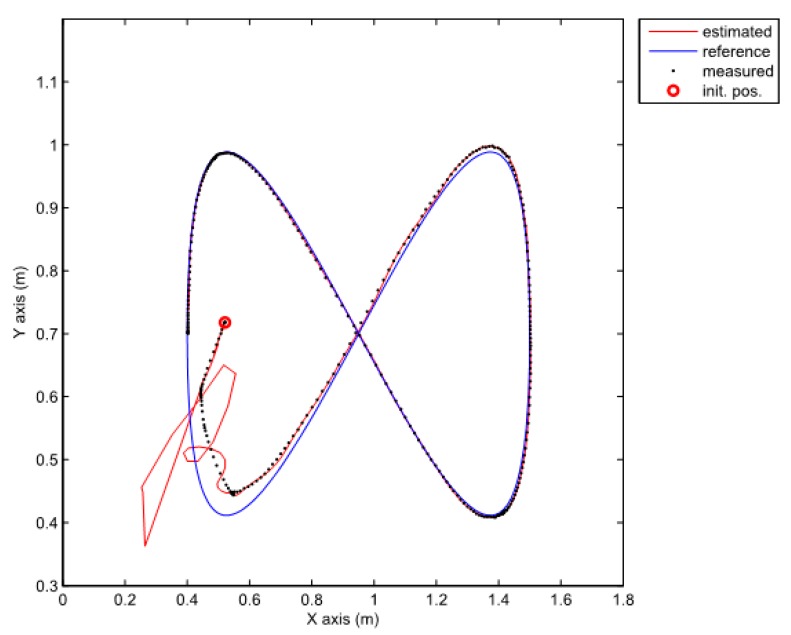
Comparison of a measured, estimated and reference trajectories in the case of the nonlinear predictor observer.

**Figure 13:**
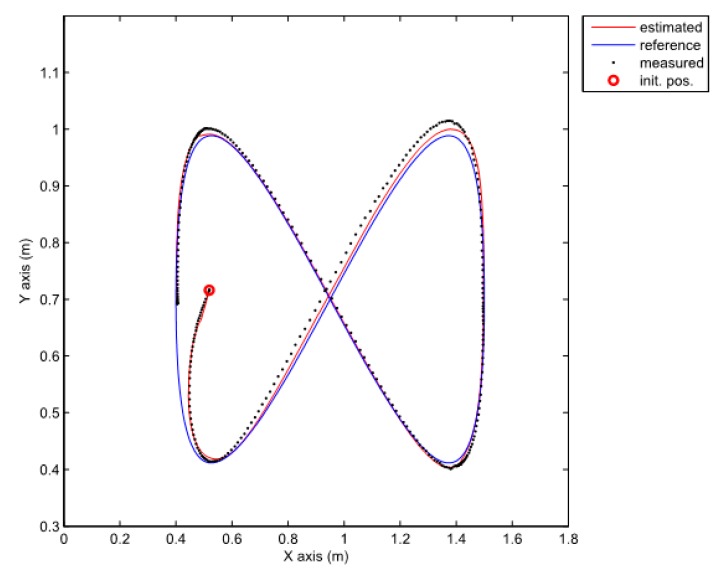
Comparison of a measured, estimated and reference trajectories in the case of the TS fuzzy predictor observer.

Note that the obtained decay rate is much better than in the previous case although all the parameters of the LMIs are the same. Two experiments were done where the above given controller is combined with the same two observers as the ones used in the previous section. We also tried to recreate the same initial pose in all cases. The posture error of the robot is shown in Figure 10, for both observers–TS refers to the observer given by Equation (25) and NL refers to the observer given by Equation (20). The robot velocities (for both observers) and the reference velocities are presented in Figure 11. The estimated trajectory of the robot (full line), the measured trajectory (dotted line) and the reference trajectory (dashed line) are presented in Figure 12, for the case of the nonlinear predictor observer and in Figure 13, for the case of the TS fuzzy predictor observer.

The approach with the PDC and the nonlinear predictor observer results in a stable behaviour and a good trajectory tracking but the initial transient is very oscillatory (see Figure 12). The approach with the PDC and the TS fuzzy predictor observer results in a stable behaviour and a better tracking of the reference trajectory with a smoother transient (see Figure 13) when compared to the results in Figure 12. Better convergence of the error states to zero in the latter case can also be noticed from Figure 10.

## 4. Discussion

In this paper, we treat the problem of trajectory tracking of a nonholonomic WMR with delayed measurements. A novel TS fuzzy predictor observer is introduced for the compensation of the measurement delay. The delay due to the vision system is known and assumed to be constant (in reality the variations are very low). The stability of the TS fuzzy predictor observer is proven and it is shown that the *L*_2_-norm of the observer error is bounded. The proposed approach with the PDC control using the TS fuzzy predictor observer is compared to a PDC control with nonlinear predictor observer. Experimental results (only a few are shown in the paper) show that the TS fuzzy predictor observer can cope with delayed noisy measurements much better than a nonlinear observer presented in [24].

In this paper, we also propose the coordinate transformation for the orientation error that helps us achieve a faster decay rate of the tracking errors. This approach extends the use of the PDC control to the cases where control errors in position are of different order of magnitude compared to the error in orientation. We tested this approach in the case where the error is given in degrees and obtained good results while the performance of the system was very poor in the absence of the coordinate transformation.

In this paper, a kinematic model of the WMR is assumed. In future work, we aim to extend this approach of control to the case of a dynamic model [37].
